# Interventional neuromodulation techniques for cervicogenic headache

**DOI:** 10.1016/j.jatmed.2025.02.003

**Published:** 2025-03-27

**Authors:** Natali Ariyoshi, Emily Qian, Rifat Abliz, Qiliang Chen

**Affiliations:** a Department of Chemistry and Biochemistry, University of Mississippi, Oxford, MS 38655, USA; b Department of Bioengineering, University of California, Berkeley, Berkeley, CA 94720, USA; c School of Medicine, West Virginia School of Osteopathic Medicine, Lewisburg, WV 24901, USA; d Department of Anesthesiology, Perioperative and Pain Medicine, Stanford University, Stanford, CA 94305, USA; e Anesthesiology Service, Veterans Affairs Palo Alto Health Care System, Palo Alto, CA 94304, USA

**Keywords:** Cervicogenic headache, Neuromodulation, Interventional techniques, Pain management

## Abstract

Cervicogenic headache is a debilitating secondary headache condition that reduces the quality of life for many. Its etiology involves pathologies in one or more of the complex cervical structures, such as cervical muscles, ligaments, facet joints, intervertebral discs, and C1–3 nerve roots. Mainstream conservative treatments, such as medication and physical therapy, are designed to address these underlying pathologies. In addition, recent advancements in neurostimulation techniques can aid in treatment-resistant or intolerant cases. This narrative review aims to critically evaluate the current treatment options for cervicogenic headaches, with a special emphasis on the efficacy of novel neuromodulation techniques and identifying their strength and limitations in treating cervicogenic headaches.

## Introduction

Headache is one of the most debilitating pain conditions, with a global prevalence of 47 %, resulting in significant reductions in quality of life and burdens to individual and societal productivity.^1^, ^2^, ^3^, ^4^, ^5^, ^6^ Moreover, headache conditions often present with overlapping signs and symptoms, making them challenging to differentiate and treat.^4^, ^7^, ^8^, ^9^ Causes of headache conditions are often complex and multifactorial. While many primary headache conditions, such as migraine or tension headache, have no clear causes, *cervicogenic headache*, in contrast, is secondary to sensitizations of various upper cervical structures and nociceptive afferents, including cervical muscles, ligaments, facet joints, intervertebral discs, and C1–3 nerve roots.^10^, ^11^, ^12^, ^13^ Further convergence of additional afferents from the trigeminal nerves with the cervical input could also lead to central sensitization of the brainstem trigeminocervical complex (TCC).^12^, ^14^, ^15^ These pathologies could originate from trauma or sex/age-related changes. For example, recent analyses revealed that 15 % of cervicogenic headache patients had experienced previous cervical injuries, such as traumatic brain injury (TBI) or whiplash injury, and 42.8 % reported the pain began immediately after the trauma.^16^, ^17^ Some evidence also suggests females are slightly more susceptible to developing cervicogenic headaches than males, with an overall prevalence of ∼4 % and the average age of onset being 32.2 years.^18^, ^19^ These degenerative changes forms the pathophysiological basis of cervicogenic headache symptoms, which allows clinicians to diagnose cervicogenic headaches based on history, physical examination, and imaging. Unlike other headache disorders, such as migraine or tension headache, cervicogenic headaches typically originate from the neck (unilateral or bilateral), affecting the high cervical, occiput, parietal, and facial regions. These headaches can be exacerbated by cervical movements, and can be alleviated with blocking cervical afferents or addressing the underlying cervical pathologies.^20^, ^21^

Pharmacological treatments for cervicogenic headaches are commonly centered on targeting the inflammatory, neuropathic, and central aspects of disease pathophysiology (Table 1). These include nonsteroidal anti-inflammatory drugs (NSAIDs), triptans, beta-blockers, anticonvulsants, and even opioids.^22^, ^23^, ^24^, ^25^, ^26^ However, long-term usage of these medications may lead to medication-overuse headaches (MOH) and dependence.^22^, ^24^, ^27^, ^28^ In addition, concurrent physical therapy (PT) is often necessary and has been shown to be beneficial for managing cervicogenic headaches by addressing the musculoskeletal co-contributors of the disease, such as relieving muscle tension, improving range of motion and correcting poor posture.^29^, ^30^, ^31^, ^32^, ^33^ Nonetheless, patient participation is essential for PT and medication management. However, the severity of the headache symptoms and the ability to tolerate treatment side effects can sometimes limit effective pain control and rehabilitation.^29^, ^31^

**Table 1:** Pharmacological treatments for headaches.

Use Case		Medication	Possible adverse effects/risks
First Line Treatment (mild to moderate headache)	**Over the Counter (OTC) Medication**	• Acetaminophen/Paracetamol	Headache, insomnia, nausea, vomiting, constipation, itching
		• Ibuprofen (NSAID)	Gastric ulcers, rash, heartburn, nausea, vomiting
		• Naproxen (NSAID)	Dizziness, headache, bruising, heartburn, gastric ulcers, gastrointestinal bleeding
		• Aspirin	Peptic ulcers, bleeding, nephrotoxicity
		• Compound Drug (Aspirin, Acetaminophen, and Caffeine)	Arrhythmias, acute liver failure, salicylate toxicity
Acute/Prophylactic Treatment (moderate to severe headache)	**Prescription Medication**	• OTC at higher doses	Same effects as listed
		• Triptans	Nausea, tight/burning sensations, adverse cardiovascular effect
		• β-blockers	Fatigue, dizziness, insomnia, depression, decreased libido
		• Anticonvulsants	Paresthesia, fatigue, weight loss, hair loss, kidney stones, cognitive problems, depression, psychosis
		• Opioids	Constipation, tolerance, high risk of dependence, addiction and medication overuse headache

For patients who are resistant or cannot tolerate conservative management, novel neuromodulation techniques could be used as either primary analgesics or adjuncts to help facilitate pain control and rehabilitation. The goal of this narrative review is to summarize and evaluate the currently available and recent advancements in neuromodulation modalities for treating cervicogenic headaches.

## Utilizing neuromodulation for treating cervicogenic headache

The mechanism of neuromodulation treatments is centered on modulating or dampening the abnormal neural activities underlying chronic headache disorders by targeting peripheral and central nociceptive pathways. These modulations of abnormal activities, in turn, reduce pain symptoms by altering the action and release of central and peripheral neurotransmitters, enhancing the spinal cord inhibitory activities and decreasing nociceptive transmission.^34^, ^35^, ^36^ Depending on the cervicogenic pathophysiology and stimulation protocols, the pain-relief effects may persist beyond the stimulation phase, leading to a reduction in both the intensity and frequency of headaches in some cases.^34^, ^36^ The following section will critically review both non-invasive and invasive neuromodulation treatment options for cervicogenic headaches (Table 2 for summary).

**Table 2:** Summary of non-invasive and invasive neuromodulation techniques for cervicogenic headache.

**Non-Invasive Methods**	**Mechanisms of Action**
• Transcranial magnetic stimulation (TMS)	Stimulation of a targeted brain region through electromagnetic induction
• Transcranial direct current stimulation (tDCS)	Stimulation of a targeted brain region through induced current
• Transcutaneous Vagus nerve stimulation (tVNS)	Stimulation of cervical or auricular branch of the vagus nerve to regulate autonomic and nociceptive transmission
• Transcutaneous supraorbital nerve stimulation (tSNS)	Stimulation of supraorbital sensory fibers to modulate trigeminal afferents
**Invasive Methods**	**Mechanisms of Action**
• Radiofrequency neuromodulation or ablation of cervical nerve roots and medial branches	Usage of electrical current to modulate afferent activities that contribute to the headache
• Greater occipital nerve stimulator implant	Stimulation of the occipital nerve/C2 afferents to modulate occipital afferents and the trigeminocervical complex (TCC) neural pathway
• Spinal cord stimulator implant	Implantation of epidural leads that target the trigeminocervical complex, disrupting nociceptive head pain signaling

### Non-invasive neuromodulation treatment options

#### Transcranial magnetic stimulation (TMS)

Transcranial Magnetic Stimulation (TMS) is a non-invasive technique involving stimulation of the brain regions through electromagnetic induction.^37^ By using an external coiled wire positioned on the scalp, a fluctuating magnetic field triggers targeted action potentials in brain regions of interest.^37^ Clinically, repetitive TMS has been used to treat chronic pain and depression by delivering repetitive pulses to modulate neural activity in the motor and dorsolateral prefrontal cortex.^38^, ^39^, ^40^, ^41^ The analgesic benefit is thought to be attributed to its action on the central nociceptive pathways and neurotransmitters, such as brain-derived neurotrophic factor (BDNF).^38^, ^39^ Intriguingly, repetitive TMS has been explored in secondary headaches after traumatic brain injuries and the associated whiplash injuries. For example, an expert panel from the International Neuromodulation Society found strong clinical evidence supporting the use of TMS on the motor cortex for headaches and craniofacial pain after TBI.^42^ Although high-quality direct evidence of TMS’s therapeutic effect on cervicogenic headaches is lacking, the close relationship between TBI and cervicogenic headaches suggests that TMS could be useful as an adjunct treatment.^43^

#### Transcranial direct current stimulation (tDCS)

Transcranial direct current stimulation (tDCS) has also been trialed in a number of primary and secondary headache conditions, including cervicogenic headaches, with some success. Stimulation of the prefrontal and motor cortex regions is believed to enhance descending pain modulation and reduce pain perception.^44^, ^45^ This stimulation modality has been shown to be beneficial in post-TBI headache and chronic migraine, reducing immediate post-treatment pain severity and the number of headache days.^46^, ^47^ The benefits of tDCSs for cervicogenic headaches appear to be derived from facilitating effective PT. Small-scale studies demonstrated that concurrent tDCS and craniocervical PT exercises resulted in a significantly greater improvement in pain level, disability index, and patient-reported comfort compared to PT alone in patients suffering from cervicogenic headaches.^48^ These positive responses prompt future ongoing double-blind randomized control trials to comprehensively assess the clinical effects of tDCS in cervicogenic headache patients, such as changes in pain, strength, function, and quality of life.^49^

#### Transcutaneous vagus nerve stimulation (tVNS)

In recent years, non-invasive cutaneous approaches to stimulate the vagus nerve have been experimented on a wide range of conditions, such as heart failure, Alzheimer's disease, obesity, tinnitus, chronic pain, and headaches.^50^, ^51^, ^52^, ^53^, ^54^, ^55^, ^56^ Transcutaneous vagus nerve stimulation (tVNS) can be achieved via either electrode or surface stimulation of the cervical vagus nerve or the auricular branch of the vagus nerve, which is thought to regulate autonomic and nociceptive transmission and, in turn, relieve pain symptoms.^57^, ^58^, ^59^, ^60^, ^61^, ^62^, ^63^, ^64^ Observational, open-label studies have shown the efficacy of non-invasive VNS in treating migraines, with pain relief achieved in 40–65 % of the total enrolled patients suffering from either chronic migraine pain or high-frequency episodic migraine (HFEM).^65^ In a recent open-label randomized controlled trial, significant reductions of headache days are observed in chronic migraine patients after being treated with tVNS in a dose-dependent manner (e.g., an average reduction of 3.9 headache days in patients who completed 4 months of tVNS, and a 7.9 headache day reduction in patients who completed 8 months of tVNS).^66^ Similar therapeutic outcomes are also observed in cluster headache treatments (e.g., headache frequency reduction from 4.5/day to 2.6/day).^67^ Although evidence of tVNS has proven to be beneficial in patients suffering from various primary headaches, studies on cervicogenic headaches are limited. However, some studies have shown the efficacy of nVNS in improving overall pain, cognition, and memory for patients with traumatic brain injuries.^68^ Given the increased prevalence of cervicogenic headaches and cervicalgia after traumatic brain injuries, potential therapeutic roles for nVNS in treating cervicogenic headaches may be established.^68^, ^69^, ^70^

#### Transcutaneous supraorbital nerve stimulation (tSNS)

Transcutaneous supraorbital neurostimulation (tSNS) is another non-invasive neuromodulation technique for chronic headache management.^71^ The underlying therapeutic mechanisms are proposed to be the result of increasing the activation threshold of trigeminal afferents, especially Aδ and C fibers.^72^, ^73^, ^74^, ^75^, ^76^ A randomized controlled trial demonstrated the efficacy and safety of tSNS in reducing migraine days, migraine effects, and total headache days in a 3-month period. Total headache days have the most significant reduction in the treatment group compared to controls (-32.7 % vs −4.1 %). Also, the long-term efficacy in headache prevention and reduction of migraine days is superior in the tSNS-treated group when compared to the sham.^77^, ^78^ Furthermore, there are observational and randomized controlled trials have demonstrated the use of supraorbital nerve stimulation in treating trigeminal neuropathic pain and supraorbital neuralgia.^79^ For example, Amin and colleagues demonstrated a 60 % pain reduction with tSNS treatment in patients suffering from supraorbital neuralgia, who also reported reductions in opioid usage at 30-week follow-up.^80^ Given the significant involvement of the trigeminal afferents in cervicogenic headache, tSNS could be a potential neuromodulation treatment option. However, further research studies are needed to establish treatment efficacy specifically for cervicogenic headache.

### Invasive neuromodulation treatment options

#### Radiofrequency neuromodulation of cervical nerve roots and branches

Direct neuromodulation of the TCC afferents, such as cervical medial branches, has long been utilized for cervicogenic headache treatment. For example, percutaneous pulsed radiofrequency ablation treatment (pRF) utilizes electrical burst current to modulate afferent activities of the cervical medial branches and nerve roots contributing to the headache.^81^ Two minutes of pRF treatment on C3–5 medial branches has been shown to be sufficient to provide significant headache relief, which was thought to be achieved by desensitizing the pathological facet joints and relaxing the cervical muscles.^82^ Supported by similar concepts, pRF on C2 dorsal root ganglion (C2-DRG), which contributes to the cervical plexus and the occipital nerves, has been applied to cervicogenic headache management with pain relief lasting up to 6 months.^83^

On the contrary, thermal radiofrequency ablation (RFA), which creates heat-induced coagulative necrosis of the targeted nerves,^84^ has also been utilized in cervical nerve roots and branches, although the efficacy for cervicogenic headache relief appears variable. Both isolated case series and systematic reviews showed significant pain relief (e.g., >50 %) immediately following cervical medial branches or nerve root RFA for cervicogenic headaches.^85^, ^86^, ^87^ However, most of these studies demonstrated a range of long-term benefits, ranging from 2 weeks to 3 months.^85^, ^86^, ^87^

#### Greater occipital nerve/C2 nerve root stimulator implant

Since pain in the occipital region is one of the defining features of cervicogenic headache, the greater occipital nerves (GON), which come off from the C2 nerve root, have been viewed as a favorable target for neuromodulation and altering the TCC pain processing.^88^, ^89^, ^90^, ^91^ Most of the clinical data for GON stimulator implants were generated for migraine and cluster headaches, with most evidence showing significant long-term clinical benefits for these conditions.^92^, ^93^, ^94^ Furthermore, a recent technical note describing a novel “Q2 approach” for C2-DRG stimulator implants demonstrated a greater than 50 % pain reduction in patients with medically refractory, intractable headaches.^95^

The clinical significance of GON stimulator implants was recently explored by Eghtesadi and colleagues in a case series of sixteen patients with moderate to severe treatment-resistant cervicogenic headaches.^96^ Notably, 11 out of 16 of these patients reported more than 50 % pain relief and quality of life improvement at the 1-year follow-up, without severe adverse effects reported by any patients at the 3-year follow-up. Five out of seven patients who were previously disabled had returned to work, supporting the use of GON stimulators in treating refractory cervicogenic headaches.^96^ However, given the multifactorial nature of cervicogenic headaches, future large-scale, high-quality studies are urgently needed to further define the stimulation parameters and patient selection before GON/C2-DRG stimulator implants can be widely adopted.

#### Spinal cord stimulator implants

Spinal cord stimulation (SCS) implant is another form of invasive neuromodulation therapy for the treatment of cervical pain.^97^ It involves the implantation of leads into the epidural space, typically in high cervical structures, to target the TCC and disrupt nociceptive signaling.^98^ Most studies involving SCS for migraines and cluster headaches, when pharmacotherapy proved insufficient, showed a near-immediate decrease in pain and intensity of attacks. This led to improvements in quality of life, demonstrating the efficacy of SCS.^97^, ^99^, ^100^ One case study from 2005 reported using SCS to treat post-traumatic cervicogenic headaches specifically and demonstrated more than 4 years of sustained pain relief with intermittent cord stimulations.^101^ However, further research should be conducted on SCS for headaches as the current literature is limited to case reports and low-quality evidence for specific headache types.

## Conclusions

The pathophysiology of cervicogenic headaches is often multifactorial, which can be attributed to pathologies in the peripheral cervical structures as well as sensitization of the central TCC nociceptive pathways. While PT and medication remain the first line of management for cervicogenic headaches, recent advancements offer viable alternatives or adjuncts for patients who cannot tolerate first-line treatment. Although both non-invasive and invasive neuromodulation treatments have shown favorable short-term benefits, the evidence is limited to small-scale case series and non-blinded randomized controlled trials. Furthermore, their respective mechanism and efficacy for different cervicogenic headache types are not well-differentiated and characterized, making it difficult to derive effective treatment algorithms for these neuromodulation techniques. To overcome these obstacles, it is thus critically important for future studies to investigate the underlying mechanisms of these neuromodulation techniques and their specific applications in patient populations with different primary headache pathologies.

## CRediT authorship contribution statement

**Natali Ariyoshi:** Writing – review & editing, Writing – original draft, Project administration, Investigation, Conceptualization. **Emily Qian:** Writing – review & editing, Writing – original draft, Visualization, Investigation. **Rifat Abliz:** Writing – review & editing, Writing – original draft, Visualization, Investigation. **Qiliang Chen:** Writing – review & editing, Writing – original draft, Supervision, Resources, Methodology, Investigation, Conceptualization. All authors have read and agreed to the published version of the manuscript.

## Consent for publication

Not applicable.

## Ethical statement

Not applicable.

## Funding

This research was supported by the VA Career Development Award IK2BX006567 from the United States (U.S.) Department of Veterans Affairs Biomedical Laboratory Research and Development Service to Qiliang Chen.

## Declaration of competing interest

The authors declare that they have no known competing financial interests or personal relationships that could have appeared to influence the work reported in this paper.

## Data Availability

All data cited in this review are publicly available through the original publications listed in the references.
